# Editorial: Cardio-vascular Dysfunction and Physiological Manifestations Induced by Environmental Conditions

**DOI:** 10.3389/fphys.2022.870917

**Published:** 2022-03-17

**Authors:** Marc-Antoine Custaud, Olga Vinogradova, Claude Gharib, Michael Delp, François Guerrero, Ronan Murphy

**Affiliations:** ^1^University of Angers, CHU Angers, CRC, INSERM, CNRS, MITOVASC, Equipe CarMe, SFR ICAT, Angers, France; ^2^Institute of Biomedical Problems, Russian Academy of Sciences, Moscow, Russia; ^3^Institut NeuroMyoGène, Université de Lyon, Lyon, France; ^4^Department of Nutrition, Food and Exercise Sciences, Florida State University, Tallahassee, FL, United States; ^5^Laboratoire ORPHY, EA 4324, Université de Bretagne Occidentale, Brest, France; ^6^Cell and Molecular Physiology Group, Faculty of Science and Health, School of Health and Human Performance, Dublin City University, Dublin, Ireland

**Keywords:** weightlessness, diving, deconditioning, gravity, extreme environment

## Introduction

Interactions between living organisms and their environment are complex, being generationally, spatially, and temporally regulated. Genetic adaptations occur and persist over many generations which confer both a survival and reproductive advantage in the context of a particular environment, a condition of evolution known as Darwinism. Acclimatization involves a constellation of integrative adaptive responses which confer functional compensation. This biological and physiological phenomenon may extend over a period of hours to months to years, even trans generationally, in response to a complex set of environmental factors. Within the bounds of the principle of parsimony, acclimation may be referred to as the functional compensation in response to a single environmental factor. Studies of acclimation are ordinarily performed in the laboratory where only one variable is manipulated at a time (Fregly, 1996). As our understanding of biological systems advance, and the contribution of growing disciplines such as epigenetics, the integrative research studies, technological platforms, modeling systems, and experimental approaches have become pivotal to elucidating functional physiological adaptations at the molecular and cellular level.

Gravity, a constant environmental condition that exists over a continuum, plays an indispensable role in maintaining normal and healthy physiological function, regulating tissue, and organ homeostasis, including that of the skeletal, muscle, neuro-vestibular, and importantly, cardiovascular system. Moreover, it plays a crucial role in the regulation of metabolism. Astonishingly, a reduction in gravitational stimuli will rapidly induce physiological and systemic de-conditioning. Over the course of the past 50 years, studies have demonstrated that the space environment and microgravity (μG) in particular, will cause physiological changes that may affect the performance and wellbeing of astronauts. These physiological changes are now better understood; prolonged exposure to a weightlessness environment (0G) can lead to significant loss of bone, muscle mass, strength, cardiovascular and sensory-motor de-conditioning, immune, hormonal, and metabolic changes. The same paradigm holds true for other equally important environmental and physiological conditions such as physical inactivity, confinement, hyperbaric, and hypoxic stimuli.

The aim of this Research Topic, in cooperation with ISGP (International Society for Gravitational Physiology—www.isgp-space.org) was to collectively gather research, studies, novel thinking, and findings from the cornucopia of work being undertaken in this field of medicine.

## Ground Models of Weightlessness

Dry immersion (DI), wet immersion, unilateral lower limb suspension, head down bed rest, and supine bed rest are ground based human models of microgravity. They induce and replicate, to various degrees, physical inactivity, and fluid transfer. As explored in their mini review (Pandianrajan and Hargens), these analogs to microgravity are complementary but should be employed cautiously and the experimental findings interrogated and interpreted objectively. Amirova et al. compared head down bed rest to dry immersion to induce cardiovascular deconditioning. Dry immersion reproduces most of the physiological effects of microgravity, importantly those such as centralization of body fluids, enhanced muscular inactivity, and support unloading. This modality of microgravity is well-understood as it was developed by Russian scientists in the early 1970s and has since been extensively employed in many studies (Navasiolava et al., 2011; Tomilovskaya et al., 2019). Support unloading is very specific to this model and induces important physiological adaptions and impairments, most notably for neuro-muscular function. Comparative studies of 21 days of head down bed rest with 3 days of dry immersion have demonstrated holistically comparable changes for the cardiovascular system. These adaptations however are more rapid and pronounced for the dry immersion model. Dry Immersion, originally developed as a model of microgravity, may also have potential therapeutic applications. For example, patients with Parkinson disease, may demonstrate and experience a beneficial effect with dry immersion. DI may not only beneficially impact and reduce muscular hypertonicity, but also potentially improves autonomic dysfunction (Megal et al.). This phenomenon is an exciting, novel, and important finding. Further translational studies utilizing and investigating the dry immersion model for therapeutic use should be actively encouraged!

## Cardiovascular Deconditioning to Microgravity

Actual and simulated weightlessness have well-described deleterious effects on the cardiovascular system. These multiple physiological adaptations and changes are termed cardiovascular de-conditioning. In their comprehensive review, Navasiolava et al. summarize the structural and functional (micro-) vascular changes induced by space flight and the various models of microgravity. Moreover, they also review the effects of prophylactic strategies and countermeasures to prevent cardiovascular de-conditioning. The endothelium, far from being an inert conduit for blood flow, is a dynamic adaptive mechanosensory tissue whose function is rapidly impaired by sedentariness and thus by weightlessness. Endothelial dysfunction may be an important arbiter of cardiovascular risk after long term exposure to microgravity.

Orthostatic intolerance is the principle acute symptom of cardiovascular deconditioning induced by weightlessness. Daily rise is a challenge for the cardiovascular system and this daily stimulation is necessary to keep the cardiovascular system reactive to gravity. Thoraco-cephalic fluid shift is a widely acknowledged consequence of weightlessness and may play a role in spaceflight associated neuro-ocular syndrome. However, our understanding of the mechanisms and physiological effects of this fluid shift is far from complete. Kirsch et al. (1984) followed and supported by Buckey et al. (1993) measured central venous pressure in astronauts and observed a decrease of central venous pressure as soon as the astronaut is exposed to 0G. Changes in the morphology of the thoracic cage, with its expansion in space environment, are very important considerations in our understanding of central venous pressure changes. Lee et al. employed a parabolic flight model to study fluid shift using ultrasound (internal jugular vein cross sectional area, inferior vena cava diameter and common carotid artery flow). Their data suggested that Gz levels >0.5 Gz are necessary to counteract, mitigate, and reduce the weightlessness induced fluid shift.

Impaired cerebral autoregulation is one of the postulated mechanisms underpinning orthostatic intolerance. However, the effects of microgravity on cerebral auto-regulation remain unclear. In their review, Kermorgant et al. considered different experimental approaches to evaluate cerebral autoregulation changes induced by spaceflight and ground-based analogs. Paradoxically, short terms studies have demonstrated a preserved or even an improved cerebral autoregulation, in contrast to long term studies, which have depicted impairment in cerebral autoregulation.

Autonomic nervous system dysfunction occurs after weightlessness exposure with an impairment of the sympathetic/para-sympathetic balance and a decrease in the cardiac baroreflex efficiency (Coupé et al., 2009). Heart rate and blood pressure variability analysis, ascertained by beat-to-beat recordings have been extensively performed both in the space environment and ground models to quantify and to explain this autonomic dysfunction. Heart rate and blood pressure changes are induced by closed loop regulation; thus, analysis of their spontaneous oscillations is particularly relevant. Borovik et al. used a phase synchronization index of beat-to-beat mean arterial pressure and heart rate to measure the baroreflex activity. The observed lack of increase of this index during head up tilt test post dry immersion corroborates a baroreflex disorder induced by weightlessness. Rusanov et al. observed changes in heart rate variability indicators after 5 days of dry immersion, indicating a shift in the autonomic balance toward a sympathetic activation. Jia et al. studied the relationship between blood perfusion in the lower extremities and hear rate variability at different positions and thus in relation with the gravity force.

## Countermeasures to Fight Deconditioning to Gravity

As physical inactivity and weightlessness induced fluid shift is the main trigger of cardiovascular deconditioning, the more obvious countermeasures are based on physical exercise and interventions to limits this phenomenon. Countermeasures based on physical exercise are acknowledged to be the most effective strategy against deconditioning, considering its pleiotropic beneficial effect on human physiology. However, since physical exercise countermeasures are not sufficient, *per se*, to fully address cardiovascular deconditioning, it is evident complementary and adjuvant strategies should be explored.

Venoconstrictive thigh cuffs are currently employed by cosmonauts to limit symptoms associated with cephalic fluid shifts. This countermeasure was tested during a 5-day dry immersion study (Robin et al.). Although wearing thigh cuffs (10 h per day) in this ground model study slowed down and mitigated loss of body water and plasma loss induced by DI, their utilization unfortunately did not prevent or counteract orthostatic intolerance.

The “Cocktail Bedrest” investigation tested the effects of an antioxidant/anti-inflammatory cocktail (polyphenol, vitamin-E, selenium, and omega-3) over a 2-month head down bed rest study. Unfortunately, this supplementation was shown to be ineffective in preventing skeletal muscle deconditioning and moreover could impair the recovery process of the muscular function and rehabilitation. This study highlights the complexity of the redox balance in the body as well as the hormesis effect and functionality of pro-oxidant molecules during long term inactivity, playing a potentially important biological role in maintaining muscle health and function (Arc-Chagnaud et al.).

However, plant extracts and antioxidant factors still need to be considered and further investigated as adjuvant countermeasures. Hesperidin, a flavonoid antioxidant component, as well as its more soluble derivative, α-Glucosyl Hesperidin, limits ankle swelling caused by a prolonged sitting position (Nishimura et al.).

It is becoming evident that the optimal countermeasure strategy will employ a stratified and multi-combinatorial approach. The MNX (Medium duration Nutrition and Resistance-Vibration Exercise) 21-day bed rest tested the combination of a nutritional supplementation using Whey proteins together with vibration and resistive exercise. Unfortunately, those countermeasures even combined failed to prevent cardiovascular deconditioning. They did however mitigate the decrease of VO_2_ max (Guinet et al.).

The “Cocktail Bedrest” and the “MNX” studies illustrate the difficulty and challenges in developing countermeasures to address the deconditioning induced by the suppression of gravity. This is due in no large part, to the fact that gravity is fundamental to health, wellbeing and normal physiological functionality of our integrated organ and tissue systems. Thus, the absence of gravity presents and remains a challenging environment to counteract and address. Extensive international studies are ongoing to test additional countermeasures (such as artificial gravity) and permutations and combinations thereof, to address the (patho-) physiological consequences to spaceflight deconditioning. Proposed long duration space flight, such as future Mars missions, will require the development of synergistic and optimal countermeasure protocols, failing which we will have no other choice but to replicate gravitational force itself using long arm rotating spaceships.

In addition to “prophylactic” strategies, “therapeutic” interventions have also been employed, since the first space flight, in the management of astronauts, cosmonauts, and taikonauts upon return to earth. Gradient compression garments (with a continuous gradient of compression from the feet until the abdomen) in the hours after long-duration spaceflight together with an end of mission fluid loading are efficient to prevent orthostatic intolerance after a 6-month space flight (Lee et al.).

## Diving Effects on the Cardiovascular Regulation

The cardiovascular system is challenged also by diving in several aspects. The first parameter is blood centralization and reduced interstitial extravasation of fluids due to both immersion and submersion. Several factors can explain this centralization, such as buoyancy, pressure gradient of hydrostatic pressure and pressure gradient between the lungs and the rest of the body. The authors (Weenink and Wingelaar) of a mini review paper argued that the compression effects on the body under immersion are not so important and could be compared to the pressure of a mild compression stocking. Although still debated (Regnard et al.—see commentary to Weenink's paper) this analysis could be of importance also for the model of dry immersion where the buoyancy effect directly counteracts gravity. Nevertheless, a direct consequence of this blood centralization is an increase in thoracic blood pressure which, in turn, leads to stimulation of the autonomous nervous system with a predominant parasympathetic activation (Schipke and Pelzer, 2001; Chouchou et al., 2009). As reported by Lundell et al. in this topic, cold exposure further increases parasympathetic activation. As highlighted by the authors, whether this potent parasympathetic activity due to the combined action of diving and cold could exert adverse effects in the case of long and cold dives merits further investigation.

A second concern, common to both diving and spaceflight, is the risk of decompression sickness (DCS) associated, respectively, to the ascent to the water surface and extravehicular activities. Currently, there is a body of literature showing that the risk of DCS after diving is decreased by nitric oxide administration whereas it is increased when NO synthesis is blocked (Wisløff et al., 2004; Dujić et al., 2006; Mazur et al., 2014). These data strongly suggest that the vascular endothelium plays a pivotal role in the development of DCS after diving, although this has never been scientifically demonstrated. The results of two studies published in this topic support this hypothesis by showing, on the one hand, that bubble formation correlates with vascular compliance and NO bioavailability (Boussuges et al.) and, on the other hand, that experimentally induced DCS in rabbits can be treated by the administration of Ulinastatin, a drug which inhibits the release of inflammatory factors and alleviates endothelial injury/dysfunction (Meng et al.).

## Necessity for Animal and Cells Models

The use of animal as well as cell models is essential for studies into environmental physiology and physiopathology.

Pressure chambers to mimic hypo- and hyperbaric conditions in rats to simulate mountain ascent or deep-sea diving were used in three articles of this Research Topic. Flores et al. investigated cardioprotective effect of caloric restriction against right ventricular hypertrophy (RVH) induced by chronic hypoxia. After chronic (30 days) of hypobaric hypoxia at 428 Tor, equivalent to that at 4,600 m above sea level, a lower degree of RVH, higher AMPK activation, and no activation of mTOR were observed in a group of rats with restricted diet compared to *ad libitum* study group. This implicates body weight as a contributing factor to RVH at high altitudes.

Gaustad et al. continuously monitored hemodynamic parameters in an intact, spontaneously breathing rat during simulated diving tests in an air-filled hyperbaric chamber during all phases of a 600-kPa dry air dive: the diving conditions were well-tolerated by a healthy heart, whereas in a failing heart, acute cardiac decompensation was recorded. The dry diving model was also employed by Ardestani et al. to study endothelium function in different vessels of the cardiovascular compartment. A single simulated dive was specifically found to induce pulmonary artery endothelial dysfunction in ApoE knockout rats, and this was more profound in male than female rats. The authors concluded that the biological mechanisms contributing to these changes were different for males and females. Translation of these findings to humans may suggest caution for divers who are elderly or have reduced endothelial function.

Hindlimb unloading (HU) in rodents is a widely used model simulating the effects of exposure to microgravity. Tarasova et al. used this model to investigate the regional and tissue specific differences in the response of skeletal muscle and cutaneous arteries to gravitational unloading. HU induced contrasting structural and functional adaptations in forelimb and hindlimb skeletal muscle arteries. Additionally, HU had diverse effects in two hindlimb vascular regions. Importantly, HU impaired sympathetically induced arterial vasoconstriction was consistent with that observed, in humans following spaceflight.

Limitations of animal models in predicting human (patho-)physiological responses and in providing clinically relevant data, is a challenge in the translation of studies. The development and use of human dynamic cellular models or “organotypic systems,” based on advanced technological platforms addresses this gap and deficit. The use of dynamic human primary cell systems coupled with emerging molecular, cellular, and (epi-)genetic technologies that allow precise control of the culture environment and analysis of meaningful endpoints paves the way for human organotypic systems as a major initiative in studying the effects of various environmental exposomes on physiological systems. Such a model was used by Liu et al. to investigate the contribution of ACE2 to mechano-transduction induced vascular remodeling under mechanical stretch and to investigate the possible underlying molecular mechanisms. Human aortic vascular smooth muscle cells (HaVSMCs) were stretched by special device *in vitro*. It was shown that mechanical stretch modulated the expression of the ACE2/Ang-(1–7) and ACE/AngII axis. ACE2 is mechano-sensitive and involved in stretch-induced dysfunction of HaVSMCs. Thus, ACE2 may contribute to the development of vascular remodeling under conditions of mechanical stretch. This observation may provide clinicians with opportunities to develop new therapeutic approaches for hypertension.

## Integrative Perspectives

### Cross Acclimation Between Ambient Oxygen and Gravity

Counter-responses adjusting for hypoxia may conflict with orthostatic responses and induce an increase of orthostatic intolerance risk. The authors (Nourdine et al.) have shown that during hypoxic orthostatic challenge, a mobilization of the heart rate reserve and a delayed systemic vascular resistance index activation are key regulatory responses to preserve orthostatic tolerance. (Boussuges et al.) studied the potential interactions between hyperoxia and exercise. If hyperoxia decreased adenosine plasma levels and increased systemic vascular resistance at rest in healthy volunteers as expected, hyperoxia did not eliminate the increase in adenosine plasma levels and in systemic vascular resistance during low intensity exercise.

### Perspectives With Proteomic Approach

As environmental conditions, especially gravity, have intrinsically complex interactions with our physiology, the use of integrative “omics” approaches in combination with data analysis is of particularly important relevance. Proteomic analysis performed on different body fluids refers to a systematic large-scale identification and quantification of proteins. Comparative analysis of different environmental exposomes can identify changes in differential protein expression and may identify novel mechanistic physiological pathways. The team of Prof. Larina, Moscow, successfully applied this method to understand mechanisms of cardiovascular changes induced by weightlessness in urine and blood samples (Pastushkova et al.; Rusanov et al.; Kashirina et al.; Kashirina et al.).

## Author Contributions

M-AC, OV, CG, MD, FG, and RM were co-editor of this Research Topic. All authors contributed to the preparation of this editorial and approved the submitted version.

## Conflict of Interest

The authors declare that the research was conducted in the absence of any commercial or financial relationships that could be construed as a potential conflict of interest.

## Publisher's Note

All claims expressed in this article are solely those of the authors and do not necessarily represent those of their affiliated organizations, or those of the publisher, the editors and the reviewers. Any product that may be evaluated in this article, or claim that may be made by its manufacturer, is not guaranteed or endorsed by the publisher.
